# *Prototheca bovis* induces autophagy in bovine mammary epithelial cells *via* the HIF-1α and AMPKα/ULK1 pathway

**DOI:** 10.3389/fimmu.2022.934819

**Published:** 2022-09-02

**Authors:** Wenpeng Zhao, Maolin Xu, Herman W. Barkema, Xiaochen Xie, Yushan Lin, Sohrab Khan, John P. Kastelic, Dong Wang, Zhaoju Deng, Bo Han

**Affiliations:** ^1^ Department of Clinical Veterinary Medicine, College of Veterinary Medicine, China Agricultural University, Beijing, China; ^2^ Department of Production Animal Health, Faculty of Veterinary Medicine, University of Calgary, Calgary, AB, Canada; ^3^ College of Life Science, Ningxia University, Yinchuan, China

**Keywords:** mastitis, *Prototheca bovis*, autophagy, bovine mammary epithelial cells, HIF-1α, AMPKα/ULK1

## Abstract

*Prototheca bovis*, a highly contagious pathogen, causes bovine mastitis, resulting in premature culling of affected cows and severe economic losses. Infection with *P. bovis* caused oxidative stress and apoptosis in bovine mammary epithelial cells (bMECs); however, mechanisms underlying *P. bovis*-induced autophagy remain unclear. Therefore, the autophagy flux induced by *P. bovis* in bMECs was analyzed by Western blot and laser scanning confocal microscopy. Expression levels of proteins in the HIF-1α and AMPKα/ULK1 pathway, including HIF-1α, AMPKα, p-AMPKα, ULK1, p-ULK1, mTOR, and p-mTOR, plus expression of autophagy-related genes including SQSTM1/p62, Atg5, Beclin1, and LC3II/LC3I, were quantified with Western blot. Infection with *P. bovis* induced autophagosomes and LC3 puncta in bMECs that were detected using transmission electron microscopy and laser scanning confocal microscopy, respectively. In addition, lysosome-associated proteins Rab7 and LAMP2a, and lysosomal activity were measured with Western blot and laser scanning confocal microscopy. Infection with *P. bovis* induced an unobstructed autophagic flux, increased protein expression of LC3II/LC3I, and decreased SQSTM1/p62 protein expression at 6 hpi. Furthermore, *P. bovis* upregulated protein expression in the HIF-1α and AMPKα/ULK1 pathway and increased the ratio of LC3II/LC3I, implying autophagy was activated in bMECs. However, deletion of AMPKα or ULK1 decreased LC3II/LC3I expression levels and LC3 puncta numbers, suggesting that autophagy was inhibited in bMECs. Additionally, deficiency of HIF-1α decreased protein expression of AMPKα and ULK1 as well as LC3 puncta numbers, and autophagy induced by *P. bovis* was also inhibited in bMECs. At 6 hpi, lysosome-associated protein Rab7 was decreased and LAMP2a was increased, indicating normal autophagy. In contrast, at 12 hpi, expression of Rab7 and LAMP2a proteins indicated that autophagy was inhibited in bMECs at that time. Therefore, we confirmed that *P. bovis* infection induced autophagy in bMECs *via* the HIF-1α and AMPKα/ULK1 pathway, with involvement of lysosome-associated protein Rab7 and LAMP2a.

## Introduction

Bovine mastitis is inflammation of bovine mammary parenchyma, most commonly caused by an infection with various pathogens. *Prototheca bovis* (*P. bovis*), an obligate heterotroph lacking chlorophyll, is a unicellular achlorophyllous algae, 3-30 µm in diameter, that lacks a specific glucosamine cell wall, and is a highly contagious pathogen causing bovine mastitis (1). *Prototheca* mastitis increases somatic cell counts and decreases milk production and milk quality, causing substantial economic losses (2, 3). Infection of bovine mammary epithelial cells (bMECs) by *P. bovis* damages cellular and subcellular organelles (4). Autophagy is an integral part of resistance to various pathogenic infections, clearing pathogens and damaged organelles (5). Infection with *P. bovis* caused oxidative stress and apoptosis in bMECs (6, 7); however, mechanisms underlying *P. bovis*-induced autophagy remains unclear.

Autophagy is a highly conserved lysosome-mediated degradation mechanism in eukaryotic cells, mainly responsible for removal of misfolded proteins, damaged organelles, etc., with an important role in maintaining cellular homeostasis (8). Autophagy can be divided into macroautophagy, microautophagy, and molecular chaperone-mediated autophagy, based on targeted transport of substrates to lysosomes (9). Macroautophagy, the best-characterized form of autophagy, involves a specialized double-membrane vesicle known as the autophagosome (10); furthermore, it is a conserved degradative pathway that host cells use to remove invading pathogens (11). During autophagy, microtubule-associated protein 1A/1B-light chain 3 (LC3-I) is lipidated to form LC3-II, an indicator of autophagic activity and flux (12). The protein SQSTM1/p62 binds to ubiquitinated substrate cargo and targets them for degradation in the autophagy-lysosome system by interacting with LC3 (13).

The unc-51 like autophagy activating kinase 1 (ULK1), a signal that initiates autophagy, has a key role in recruitment of autophagy-related genes and proteins to phagocytic vesicle assembly sites (14). ULK1 is the key mediator of mTORC1 signaling to autophagy. In the presence of amino acids, mTORC1 is active and inhibits autophagy by phosphorylating ULK1 and ATG13. However, when nutrients are deficient, mTORC1 activity on the surface of lysosomes is inhibited, and ULK1 and ATG13 are rapidly dephosphorylated, leading to activation of ULK1 kinase and induction of autophagy (15). Additionally, AMP activated protein kinase (AMPK), a key energy sensor, can also activate ULK1 activity by phosphorylating ULK1 during nutrient energy deprivation, thereby further promoting autophagy (16). High mTOR activity prevents ULK1 activation by phosphorylating ULK1 and disrupting interactions between ULK1 and AMPK (17). Hypoxia-inducible factor-1α (HIF-1α) is upregulated by AMPK activation and mtROS and is required for expression of anti-inflammatory genes and induction of autophagy (18, 19). In addition to hypoxia, HIF-1α can also be activated in nonhypoxic conditions in many cell types (19). Activation of HIF-1α also involved in autophagy. Deficiency of HIF-1α enhances influenza A virus replication by promoting autophagy in alveolar type II epithelial cells (20). Additionally, lysosomal-associated membrane protein 2 (LAMP2) and ras associated protein 7 (Rab7, a member of the small GTPase family), have important roles in promoting integrity of the lysosome membrane and in regulating fusion of autophagy vesicles and lysosomes (21).

Autophagy is closely related to resistance to various pathogenic infections and clearance of pathogens and damaged intracellular organelles (5, 22); however, mechanisms underlying autophagy in bMECs caused by *P. bovis* infection remain unclear. Therefore, in this study, we established a model of autophagy induced by *P. bovis* infection of bMECs and demonstrated that *P. bovis* infection induced autophagy *via* the HIF-1α and AMPKα/ULK1 pathway, with involvement of lysosome-associated protein Rab7 and LAMP2a in bMECs.

## Materials and methods

### *Prototheca bovis* strains

Isolates of *Prototheca bovis* recovered from clinical mastitis milk samples on Chinese dairy farms were stored at 4°C in the College of Veterinary Medicine, China Agricultural University, Beijing, China (7). These isolates were multiplied by streaking on sabouraud dextrose agar (SDA) and incubating the plate at 37°C for 48 h. Then, a single colony was placed in sabouraud dextrose broth (SDB) and incubated for 72 h. Thereafter, organisms were diluted in DMEM to pre-defined concentrations.

### Cell culture and infection

The MAC-T cells (Shanghai Jingma Biological Technology Co., Ltd. China) was cultured for use in various experiments. The bMECs were placed in DMEM medium supplemented with 10% fetal bovine serum, penicillin (100 U/mL) and streptomycin (100 U/mL), and grown in cell culture plates in 5% CO_2_ at 37°C. Cells from passages 2-8 were used for experiments. Before infection, cells were seeded in 6-well plates (1×10^5^ cells per well) and cultured for 24 h. Then, we changed to a new medium (without penicillin or streptomycin), the bMECs were infected with *P. bovis* at a 5:1 multiplicity of infection (MOI; ratio of *P. bovis* to bMECs) and incubated in 5% CO_2_ at 37°C for 6 h. Thereafter, samples were collected and proteins extracted and used for Western blots. The experiment was repeated 3 times to ensure reproducibility.

### Cell transfection and confocal microscope observation

The bMECs were seeded in 6-well plates and grown to 50% confluence. Ad-mCherry-GFP-LC3B (Beyotime, #C301) was used to transfect bMECs at a 20:1 MOI for 12 h in DMEM containing 10% FBS, following manufacturer’s instructions. Then, bMECs were infected with *P. bovis* at a 5:1 MOI for 0, 2, 4, 6, 8, 10, or 12 h. Additionally, rapamycin (20 µM; MCE, #AY-22989) and 3-Methyladenine (5 mM; MCE, #HY-19312) were used to treat cells for 6 h as an inducer and inhibitor of autophagy, respectively. Cell nuclei were stained with Hoechst 33342 (Beyotime, #C1025), washed 3 times with PBS, and observed with laser scanning confocal microscopy (Nikon, A1 LFOV) at laser wavelengths of 405, 488 and 561 nm. In addition, Ad-GFP-LC3B (Beyotime, #C3006) was also used to transfect bMECs at a 20:1 MOI for 12 h in DMEM containing 10% FBS. The bMECs were infected with *P. bovis* at a 5:1 MOI for 6 h. Cell nuclei were stained with Hoechst 33342, and after 3 PBS washes, cells were examined under a laser scanning confocal microscope (Nikon, A1 LFOV) at laser wavelengths of 405 and 488 nm.

### Transmission electron microscopy

The bMECs (1×10^5^ cells per well) were seeded in 6-well plates, grown to 80% confluence and infected with *P. bovis* at 5:1 MOI or treated with rapamycin (20 µM) and 3-Methyladenine (5 mM) for 6 h. For transmission electron microscopy, samples were prepared according to our previous method (23). Cells were washed 3 times with PBS, fixed with 2.5% glutaraldehyde for 4 h at room temperature, then post-fixed in 0.5% osmium tetroxide for 2 h. Samples were dehydrated in an ethanol gradient, followed by acetone (15 min in each solution). Thereafter, samples were embedded in resin and thin slices (100 nm) were cut with a glass knife. Sections were put on copper grids, stained with 2% uranyl acetate and lead citrate, and examined with a transmission electron microscope (H7650, Tokyo, Japan) at an accelerating voltage of 80 kV.

### siRNA transfection

The bMECs were seeded into 6-well plates; when the cells had achieved 40-50% confluence, they were transiently transfected with siRNA by Lipo8000™ transfection reagent (Beyotime, #C0533), according to the manufacturer’s instructions. For cells in 1 well of the 6-well plate to be transfected, 100 pmol siRNA was added to 125 µL DMEM medium without antibiotics or serum, and mixed by pipetting gently; then 4 µL Lipo8000™ Transfection Reagent was added, mixed by gentle pipetting, and incubated at room temperature for 20 min. After transfection for 6 h, fresh medium was added into wells and transfected cells were cultured for another 24 h. The efficiency of siRNA transfection was determined with Western blot. The following siRNAs were used: HIF-1α siRNA, sense (5’-3’) GGAUAUGUCUGGAUAGAAATT, antisense (5’-3’) UUUCUAUCCAGACAUAUCCTT; ULK1 siRNA, sense (5’-3’) GCUUCAGCGCAACCACAAATT, antisense (5’-3’) UUUGUGGUUGCGCUGAAGCTT; AMPKα siRNA, sense (5’-3’) GCUGGUCCAGAGGUAGAUATT, antisense (5’-3’) UAUCUACCUCUGGACCAGCTT; NCsiRNA, sense (5’-3’) UUCUCCGAACGUGUCACGUTT, antisense (5’-3’) ACGUGACACGUUCGGAGAATT. All siRNAs used were synthesized by Sangon Biotech (Shanghai) Co., Ltd.

### Western blot

The bMECs were treated as described above, lysed on ice for 5 min, and the cell lysate suspensions were collected and centrifuged at 12000×g for 15 min at 4°C. Total protein concentration in the supernatant was determined with a BCA protein assay kit, according to the manufacturer’s instructions (Beyotime, #P0012S). Protein samples were mixed with SDS-PAGE protein loading buffer (5×), denatured in boiling water for 10 min, then separated by SDS-PAGE and transferred onto polyvinylidene difluoride membranes. These membranes were blocked with 5% nonfat dry milk for 2 h at room temperature, then incubated overnight at 4°C with the following primary antibodies: β-actin (Abcam, #4970, 1:1000), HIF-1α (Abcam, #ab179483, 1:1000), ULK1 (Proteintech, #20986-1-AP, 1:1000), phospho-ULK1 (Ser 757: CST, #14202, 1:1000; Ser 556: Affinity Biosciences, #DF7587, 1:1000; Ser 317: Affinity Biosciences, #AF2301, 1:1000), AMPKα (CST, #5831, 1:1000), phospho-AMPKα (CST, #2535, 1:1000), mTOR (Proteintech, #28273-1-AP, 1:1000), phospho-mTOR (Proteintech, #67778-1-Ig, 1:1000), Atg5 (Abcam, #ab228668, 1:1000), LC3 (Proteintech, #14600-1-AP, 1:1000), Beclin1 (Abcam, #ab231341, 1:1000), SQSTM1/p62 (Proteintech, #118420-1-AP, 1:1000), LAMP2a (Proteintech, #66301-1-Ig, 1:1000), and Rab7 (Abcam, #ab229647, 1:1000). Thereafter, they were incubated with secondary antibody HRP-conjugated affinipure goat anti-rabbit IgG (H+L) (Proteintech, #SA00001-2, 1:5000) for 1 h at room temperature. After washing with Tris-buffered saline, the membrane was developed using ECL reagents and visualized with a chemiluminescence system. Results were normalized to β-actin, and band density assessed with Image J software (Version 1.8.0, National Institutes of Health, Bethesda, MD, USA).

### Immunofluorescence

After infecting bMECs with *P. bovis* for 6 h in 6-well plates, bMECs were washed 3 times with PBS, fixed in 4% paraformaldehyde for 4 h, and subsequently permeabilized in 0.25% Triton X-100 (Beyotime, #ST797) for 15 min. Cells were incubated with 3% bovine serum for 30 min at room temperature and then incubated at 4°C overnight with primary antibody ULK1 (Proteintech, #20986-1-AP, 1:500). Next, cell samples were washed 3 times with PBS and incubated with Alexa Fluor 488-labeled goat anti-rabbit IgG (H+L) (CST, #8878, 1:500) for 1 h at room temperature, then washed 3 times with PBS and stained with DAPI (Beyotime, #C1002) for 5 min. After washing with PBS, slides were covered with glass cover slips and ULK1, examined with a laser scanning confocal microscope (Nikon, A1 LFOV) at laser wavelengths of 405 and 488 nm, and images captured were analyzed with Image J software (Version 1.8.0).

### Lyso−tracker red staining

Lyso-Tracker Red, a lysosome red fluorescent probe that penetrates cell membranes, is used to detect acidification of lysosomes or mature autophagosomes in living cells. Ad-GFP-LC3B (Beyotime, #C3006) was used to transfect bMECs at a 20:1 MOI for 12 h in DMEM containing 10% FBS. After infecting bMECs with *P. bovis* for 6 h, cell culture medium was removed, cells were washed 3 times, and then incubated with Lyso-Tracker Red (50 nM) (Beyotime, #C1046) at 37°C for 30 min. The Lyso-Tracker Red staining solution was removed, and fresh cell culture solution was added, followed by examination with a laser scanning confocal microscope (Nikon, A1 LFOV) at laser wavelengths of 405, 488 and 561 nm.

### Statistical analyses

All data were analyzed by Student’s *t*-test or one-way ANOVA with Bonferroni correction for multiple comparisons. For all analyses, *P* < 0.05 was considered significant in a 2-tailed statistical test. Data were reported as mean ± standard deviation (SD) of 3 independent experiments.

## Results

### Autophagy in bMECs induced by *P*. *bovis* infection

**Figure 1:**
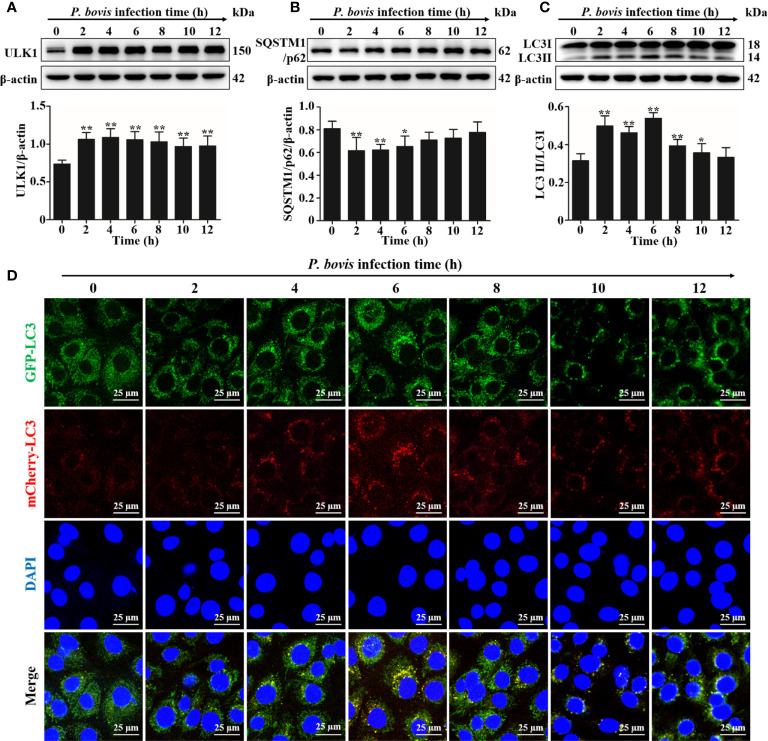
P*. bovis* infection induced autophagy in bMECs. **(A)** Protein expression of LC3II/LC3I and SQSTM1/p62 in bMECs continuously infected with *P. bovis* for 12 h **(B)** and **(C)** Quantification of LC3II/LC3I and SQSTM1/p62 proteins by ImageJ software. **(D)** Autophagic flux in bMECs. Cells were transfected with Ad-mCherry-GFP-LC3 (MOI=20:1) for 12 h, and then infected with *P. bovis* for various intervals (0, 2, 4, 6, 8, 10, and 12 h) to monitor the autophagic flux with laser scanning confocal microscopy. Data represent means ± SD of 3 independent experiments, compared to the control group (*P < 0.05 and **P < 0.01).

Quantification of changes in LC3-II and SQSTM1/p62 are widely used to monitor autophagy. To investigate the autophagy induced by *P*. *bovis* infection in bMECs, protein expression levels of ULK1, SQSTM1/p62 and LC3II/LC3I were assessed at various time points. Expression of ULK1 was increased after *P*. *bovis* infection in bMECs (**Figure 1A**). Infection with *P*. *bovis* decreased protein expression level of SQSTM1/p62 compared to level of protein in uninfected groups at 2, 4 and 6 hpi (**Figure 1B**). Protein expression level of SQSTM1/p62 had an upward trend at 8, 10, and 12 hpi, albeit without a significant difference (**Figure 1B**). Meanwhile, protein expression of LC3II/LC3I significantly increased compared to the uninfected group at 2, 4 and 6 hpi, suggesting autophagy had occurred during *P. bovis* infection in bMECs; however, it subsequently decreased at 8 and 10 hpi (**Figure 1C**, **Figure 1SA**), with no significant difference between expression level of protein in LC3II/LC3I and the uninfected group at 12 hpi (**Figure 1C**, **Figure 1SA**), implying that autophagy was inhibited or blocked. Adenovirus expressing mCherry-GFP-LC3B fusion protein is an adenovirus expressing mCherry-GFP-LC3B (Ad-mCherry-GFP-LC3B) fusion protein that can be used to detect autophagy after infecting cells. bMECs were continuously infected with *P*. *bovis* for 12 h to examine the dynamics of autophagy. Autophagolysosome fluorescence tended to increase within 6 h, along with duration of infection, whereas red mottled fluorescence increased, peaking at 12 h (**Figure 1D**), implying that autophagy was unobstructed for 6 hpi, but was subsequently inhibited or blocked in bMECs infected with *P. bovis*.

### *P. bovis* infection activated autophagy by modulating the AMPKα/ULK1 pathway in bMECs

**Figure 2:**
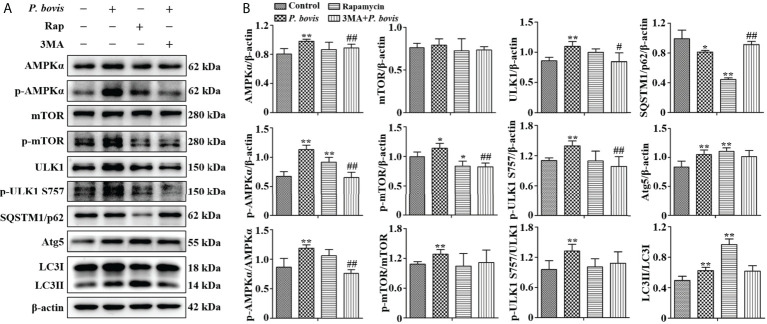
P*. bovis* infection induced autophagy *via* activated AMPKα/ULK1 signaling in bMECs. **(A)** and **(B)** Western blot analyses of AMPKα, p-AMPKα, p-mTOR, ULK1, p-ULK1, SQSTM1/p62, Atg5 and LC3II/LC3I in bMECs. The right panel is protein quantification with ImageJ software. The bMECs were infected with *P. bovis* and treated with rapamycin (20 µM) for 6 hAdditionally, bMECs were pretreated with 3MA (5 mM) for 2 h, and then infected with *P. bovis* for 6 h Data represent means ± SD of 3 independent experiments, compared to the control group, *P < 0.05 and **P < 0.01; compared to the P. bovis group, ^#^ *P* < 0.05 and ^##^ *P* < 0.01.

The AMPKα/ULK1 pathway is involved in regulation of autophagy. To measure activated autophagy in bMECs infected with *P. bovis*, protein expression levels in AMPKα/ULK1 were measured 6 h after *P. bovis* infection. Protein expression levels of HIF-1α, p-AMPKα, p-mTOR and p-ULK1(ser 757, 317 and 556) were increased after *P. bovis* infection (**Figures 2A, B**, **Figure 2S**), with no significant difference in protein expression between the mTOR and the control group. Elevated phosphorylation protein levels implied that the AMPKα/ULK1 pathway was activated by *P. bovis*. Furthermore, protein expression of autophagy-related genes was also examined, and protein expression level of SQSTM1/p62 was decreased, whereas expression of Atg5 and LC3II/LC3I was increased in bMECs after *P. bovis* infection (**Figures 2A, B**, **Figure 1SB**). Rapamycin increased protein expression of p-AMPKα, p-ULK1(ser 757, 317 and 556), Atg5, and LC3II/LC3I, but decreased p-mTOR and SQSTM1/p62 protein expression levels in bMECs compared to the control group (**Figures 2A, B**, **Figure 1SB** and **Figure 2S**). Pretreatment with 3MA decreased protein expression levels of AMPKα, p-AMPKα, p-mTOR, ULK1, p-ULK1(ser 757, 317 and 556) and LC3II/LC3I, but increased SQSTM1/p62 protein expression in bMECs compared to the *P. bovis* infection group (**Figures 2A, B**, **Figure 1SB** and **Figure 2S**). The activated AMPKα/ULK1 pathway was involved in *P. bovis*-induced autophagy in bMECs.

### *P. bovis* infection induced autophagosome formation in bMECs

**Figure 3:**
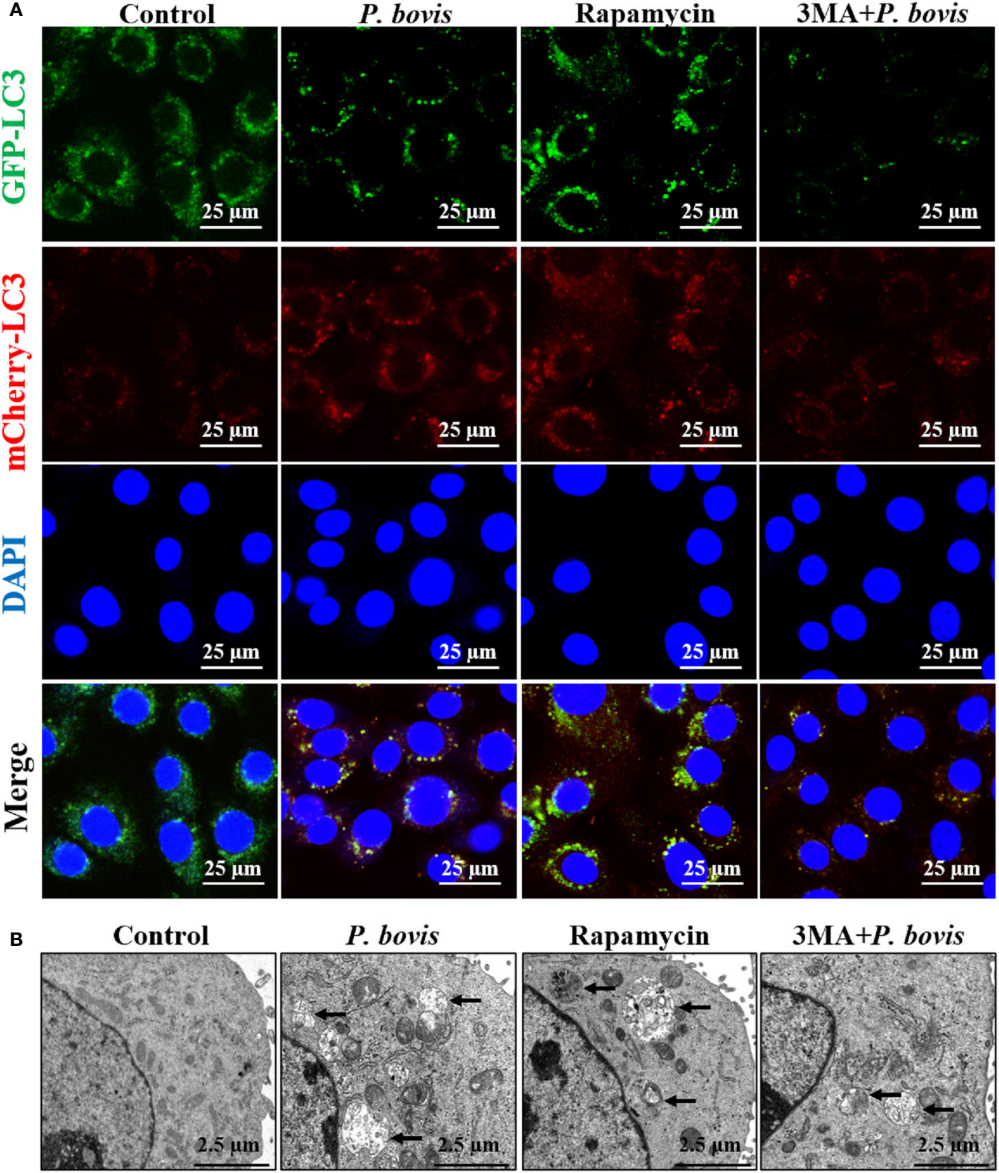
P*. bovis* infection induced autophagosome formation in bMECs. **(A)** Measurement of autophagosomes in bMECs. Cells were infected with *P. bovis* and treated with rapamycin (20 µM) or 3MA (5 mM) for 6 h **(B)** Ultrastructural observation after bMECs infection with *P. bovis*, treatment with rapamycin or 3MA for 6 h Black arrows indicated autophagosomes.

Induction of autophagy is formation of an autophagosome. Based on cells transfected with Ad-mCherry-GFP-LC3, *P. bovis* infection induced autophagosome formation in bMECs, as indicated by green and yellow puncta in the *P. bovis* infection and rapamycin treatment group (**Figure 3A**). Meanwhile, autophagosomes were also observed in the *P. bovis* infection and rapamycin treatment group (**Figure 3B**). Pretreatment with 3MA inhibited autophagosome formation in *P. bovis*-infected bMECs, although some autophagosomes were observed in these bMECs (**Figures 3A, B**). Therefore, *P. bovis* infection induced autophagosome formation in bMECs.

### AMPKα deletion reduced ULK1 phosphorylation and also inhibited autophagy in bMECs infected with *P. bovis*

**Figure 4:**
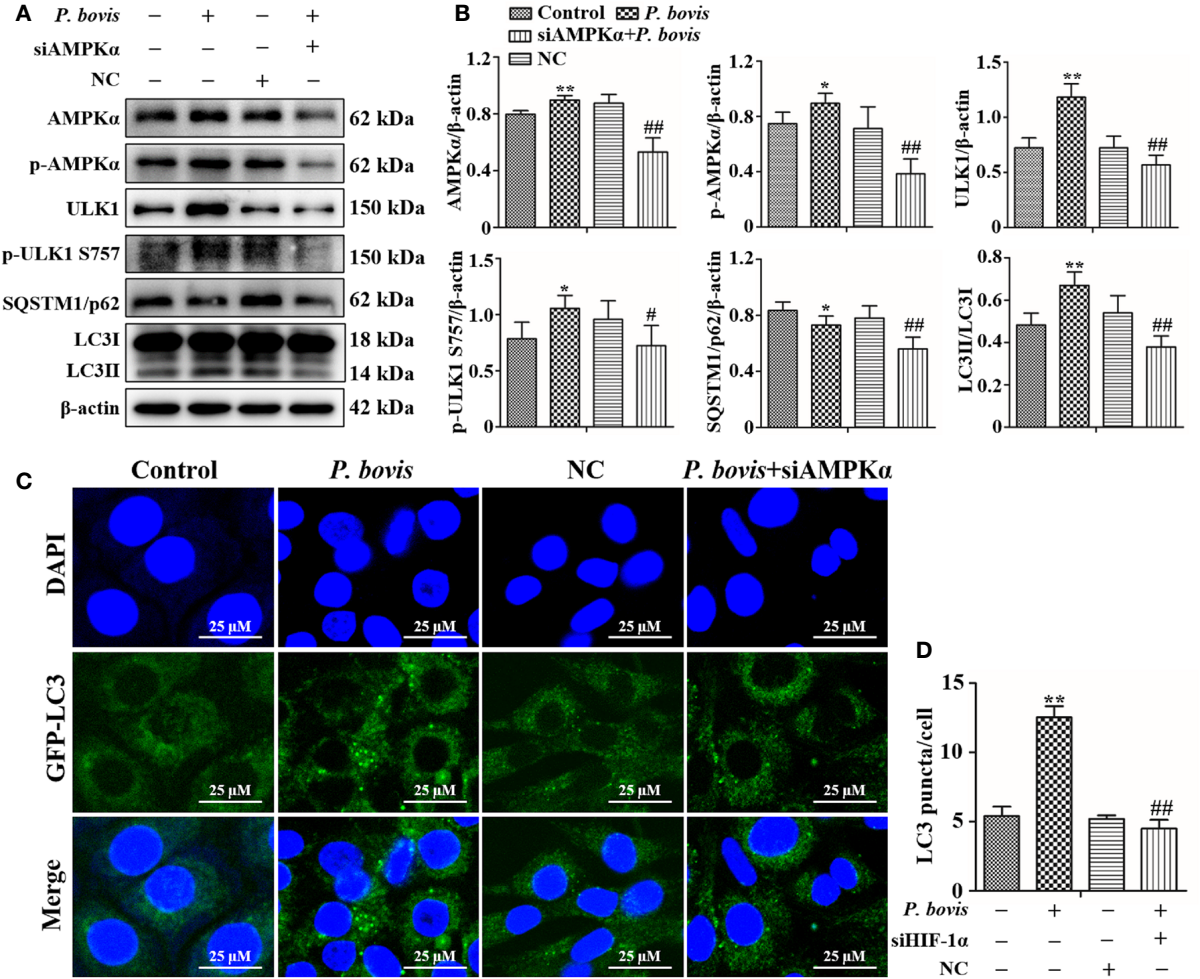
Silencing AMPKα reduced *P. bovis*-induced autophagy in bMECs. **(A)** Western blot analysis of AMPKα, p-AMPKα, ULK1, p-ULK1, SQSTM1/p62 and LC3II/LC3I in bMECs. The right panel indicates protein quantification (ImageJ software). **(B)** Autophagosome measurement in *P. bovis-* infected bMECs. First, Ad-GFP-LC3B was used to transfect bMECs at a 20:1 multiplicity of infection for 12 h in DMEM containing 10% FBS; thereafter, bMECs was infected with *P. bovis* for 6 h NC indicated non-target siRNA control. Data represent means ± SD of 3 independent experiments, compared to the control group, *P < 0.05 and **P < 0.01; compared to the P. bovis group, ^#^ *P* < 0.05 and ^##^ *P* < 0.01.

**Figure 5:**
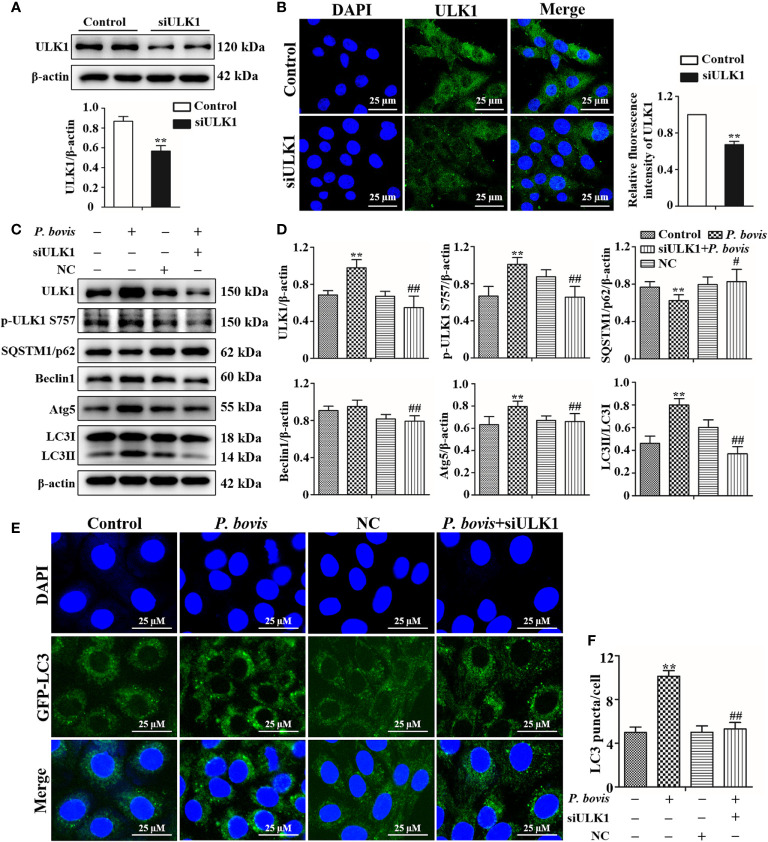
Silencing ULK1 reduced *P. bovis* induced autophagy in bMECs. **(A)** and **(B)** Western blot and immunofluorescence analyses of ULK1 after *P. bovis* infection for 6 h in bMECs. The right panel is protein quantification by ImageJ software. **(C)** and **(D)** Western blot analyses of ULK1, p-ULK1, SQSTM1/p62, Beclin1, Atg5, LC3I, and LC3II after *P. bovis* infection for 6 h in bMECs. The right panel indicates protein quantification by ImageJ software. **(E)** and **(F)** Autophagosome measurement in *P. bovis* infection bMECs. Ad-GFP-LC3B was used to transfect bMECs at a 20:1 multiplicity of infection for 12 h in DMEM containing 10% FBS; thereafter, bMECs were infected with *P. bovis* for 6 h NC indicated non-target siRNA control. Data represent means ± SD of 3 independent experiments, compared to the control group, **P < 0.01; compared to the P. bovis group, ^#^ *P* < 0.05 and ^##^ *P* < 0.01.

AMPKα has an important role in regulation of autophagy. To verify the role of AMPKα in *P. bovis*-induced autophagy in bMECs, we silenced AMPKα in cells. Infection with *P. bovis* increased protein expression levels of AMPKα, p-AMPKα, ULK1, p-ULK1 and LC3II/LC3I, but decreased SQSTM1/p62 protein expression in bMECs compared to the control group (**Figures 4A, B**, **Figure 1SC**), suggesting that autophagy induced by *P. bovis* was activated in bMECs. However, silencing AMPKα decreased p-AMPKα protein expression; furthermore, protein expression levels of HIF-1α, ULK1, p-ULK1 (ser 757, 317 and 556), SQSTM1/p62, and LC3II/LC3I were also decreased during *P. bovis* infection in bMECs (**Figures 4A, B**, **Figure 1SC**, **Figure 3S**). Therefore, we inferred that deletion of AMPKα inhibited autophagy induced by *P. bovis*. Additionally, deletion of AMPKα did not affect protein expression of HIF-1α during *P. bovis* infection (**Figure 3S**), and addition of non-target siRNA did not affect protein expression in *P. bovis-*infected bMECs (**Figure 4S**). Meanwhile, *P. bovis* infection increased the number of LC3 puncta (average of ~12.5 puncta per cell), deletion of AMPKα also reduced the number of LC3 puncta (average of ~4.5 puncta per cell) in *P. bovis*-infected bMECs (**Figures 4C, D**), and the fluorescence intensity of total GFP-LC3B was also decreased in bMECs (**Figure 5SA**). Deletion of AMPKα reduced ULK1 phosphorylation and inhibited *P. bovis* infection induced autophagy in bMECs, but did not affect protein expression of HIF-1α in bMECs.

### Deletion of ULK1 reduced *P. bovis* infection induced autophagy in bMECs

**Figure 6:**
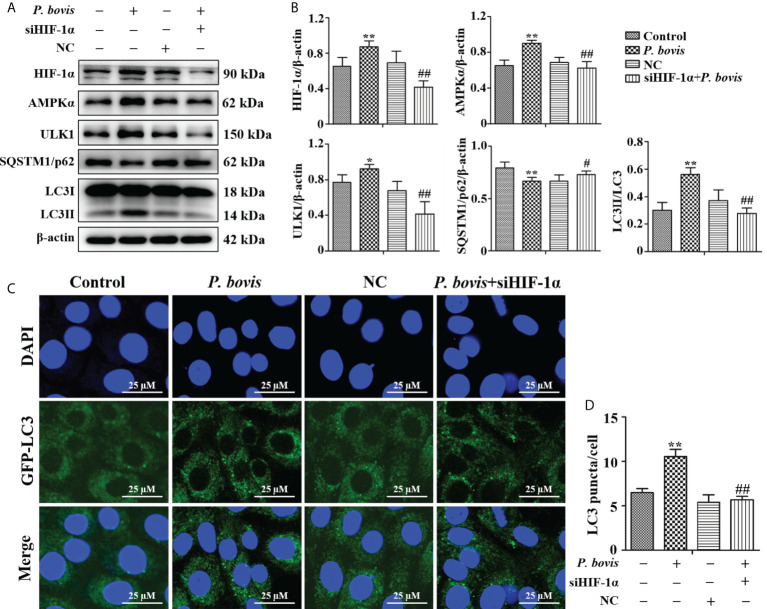
HIF-1α deletion reduced *P. bovis* induced autophagy in bMECs. **(A)** and **(B)** Western blot analyses of HIF-1α, AMPKα, ULK1, SQSTM1/p62, LC3I, and LC3II in bMECs after *P. bovis* infection for 6 h The right panel indicates protein quantification by ImageJ software. **(C)** Autophagosome measurement in *P. bovis* infection bMECs. Ad-GFP-LC3B was used to transfect bMECs at a 20:1 multiplicity of infection for 12 h in DMEM containing 10% FBS; then, bMECs were infected with *P. bovis* for 6 h NC indicated non-target siRNA control. Data represent means ± SD of 3 independent experiments, compared to the control group, *P < 0.05 and **P < 0.01; compared to the P. bovis group, ^#^ *P* < 0.05 and ^##^ *P* < 0.01.

ULK1 is a key gene for autophagy initiation; therefore, we examined effects of ULK1 deletion on the induction of autophagy in bMECs by *P. bovis*. Protein expression of ULK1 was significantly decreased in bMECs after silencing ULK1 (**Figure 5A**). Furthermore, the green fluorescence of intensity of ULK1 was also decreased compared to the control group (**Figure 5B**). These results suggested that the ULK1 gene was silenced. *P. bovis* infection increased protein expression levels of ULK1, p-ULK1(ser 757, 317 and 556), Atg5 and LC3II/LC3I in bMECs compared to the control group (**Figures 5C, D**, **Figure 1SD** and **Figure 6S**); although protein expression of Beclin1 was also increased, it was not significant. ULK1 deletion decreased protein expression levels of p-ULK1(ser 757, 317 and 556), Beclin1, Atg5, and LC3II/LC3I, whereas SQSTM1/p62 protein expression level was increased in bMECs infected with *P. bovis* (**Figures 5C, D**, **Figure 1SD** and **Figure 6S**). Therefore, protein expression levels were affected by deletion of ULK1 in *P. bovis-*infected cells. Meanwhile, *P. bovis* infection increased protein expression of HIF-1α and AMPKα, and deletion of ULK1 did not affect protein expression of HIF-1α and AMPKα in the *P. bovis*-infected group (**Figure 6S**). Furthermore, the addition of non-target siRNA did not affect protein expression in *P. bovis-*infected bMECs (**Figure 4S**). Additionally, *P. bovis* infection increased the number of LC3 puncta (average of ~10.1 puncta per cell), whereas silencing ULK1 reduced the numbers of LC3 puncta (average of ~5.3 puncta per cell) in bMECs infected with *P. bovis* (**Figures 5E, F**). In addition, the fluorescence intensity of total GFP-LC3B was also decreased in bMECs (**Figure 5SB**). These results suggested that ULK1 deletion reduced *P. bovis* infection-induced autophagy in bMECs.

### HIF-1α involved in *P. bovis* induced autophagy in bMECs

**Figure 7:**
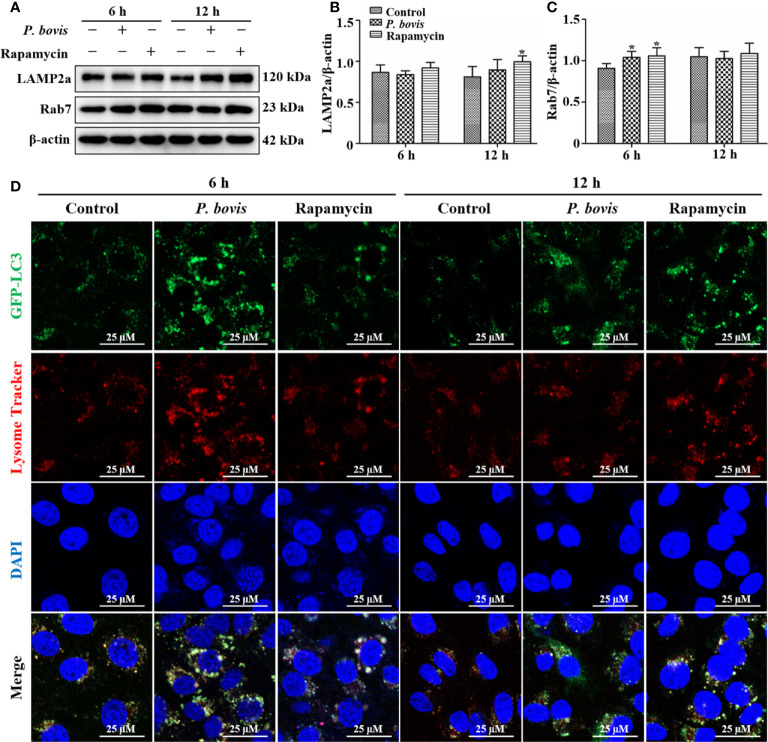
Autophagolysosome observations in bMECs. **(A–C)** Western blot analysis of LAMP2a and Rab7 in bMECs. The right panel indicates protein quantification by ImageJ software. The bMECs were infected with *P. bovis* and treated with rapamycin (20 µM) for 6 and 12 h, respectively. **(D)** Autophagolysosome measurement in *P. bovis* infection bMECs. Ad-GFP-LC3B was used to transfect bMECs at a 20:1 multiplicity of infection for 12 h in DMEM containing 10% FBS; then, bMECs were infected with *P. bovis* and treatment with rapamycin (20 µM) for 6 and 12 h, respectively, and incubated with Lyso-Tracker Red (50 nM) at 37°C for 30 min. Data represent means ± SD of 3 independent experiments, compared to the control group (*P < 0.05).

To examine whether HIF-1α is involved in *P. bovis*-induced autophagy of bMECs, we silenced the HIF-1α gene and then measured effects on the AMPK/ULK1 pathway and autophagy. *P*. *bovis* infection increased protein expression levels of HIF-1α, AMPKα and ULK1, but decreased SQSTM1/p62 protein expression in bMECs (**Figures 6A, B**). However, silencing HIF-1α reduced AMPKα, ULK1, ULK1 and LC3II/LC3I expression, and it also increased SQSTM1/p62 protein expression (**Figures 6A, B**, **Figure 1SE**), which reduced autophagy induced by *P. bovis*. The protein expression levels of p-AMPKα and p-ULK1(ser 757, 317 and 556) were decreased after deletion of HIF-1α during *P. bovis* infection (**Figure 7S**). Furthermore, the addition of non-target siRNA did not affect protein expression in *P. bovis-*infected bMECs (**Figure 4S**). Meanwhile, *P. bovis* infection increased the number of LC3 puncta (average of ~10.5 puncta per cell), whereas silencing HIF-1α reduced the number of LC3 puncta (average of ~5.7 puncta per cell) compared to the *P. bovis* infection group (**Figures 6C, D**). Furthermore, fluorescence intensity of total GFP-LC3B was also decreased in bMECs (**Figure 5SC**). These results suggested that HIF-1α was involved in *P. bovis*-induced autophagy, and that deletion of HIF-1α inhibited autophagy in bMECs *via* the AMPKα and ULK1 pathway.

### *P. bovis* infection destroyed lysosomes and inhibited autophagy in bMECs

In the late stage of autophagy, mature lysosomes fuse with autophagosomes to form autophagolysosomes to degrade damaged organelles or pathogens, with LAMP2a and Rab7 being involved in lysosome maturation. Protein expression of LAMP2a was decreased at 6 hpi, but increased at 12 hpi in bMECs infected with *P. bovis*, albeit not significantly, as compared to the control group (**Figures 7A, B**). Furthermore, rapamycin treatment increased protein expression of LAMP2a in bMECs at both 6 and 12 hpi (**Figures 7A, B**). Protein expression of Rab7 was increased at 6 hpi, but decreased at 12 hpi in bMECs, compared to the control group (**Figures 7A, C**). Furthermore, rapamycin treatment increased protein expression of Rab7 in bMECs at 6 and 12 hpi (**Figures 7A, C**). We inferred that the normal expression of LAMP2a and Rab7 was involved in maturation of lysosomes at 6 hpi, but lysosomes were destroyed at 12 hpi. In bMECs incubated with Lyso-Tracker Red and Ad-GFP-LC3B, red fluorescence was increased around intracellular LC3 puncta in the *P. bovis* infection and rapamycin treatment group at 6 h (**Figure 7D**). However, the red fluorescence was reduced around intracellular LC3 puncta at 12 h, although the red fluorescence in rapamycin treatment was stronger than in the *P. bovis* infection group at 12 h (**Figure 7D**). We inferred that *P. bovis* infection destroyed lysosomes, which inhibited autophagy in bMECs.

## Discussion

*Prototheca bovis* infection induced autophagy flux, increased protein expression of LC3II/LC3I and decreased SQSTM1/p62 protein expression, which activated autophagy in bMECs. However, silencing AMPKα or ULK1 decreased LC3II/LC3I protein expression and inhibited autophagy in bMECs induced by *P. bovis*, whereas deletion of HIF-1α decreased protein expression levels of AMPKα and ULK1 and inhibited *P. bovis*-induced autophagy. Additionally, the decrease in LAMP2a and increase in Rab7 indicated that *P. bovis* induced autophagy in bMECs. Therefore, we concluded that *P. bovis* infection induced autophagy *via* the HIF-1α and AMPKα/ULK1 pathway in bMECs and that lysosome-associated protein Rab7 and LAMP2a were also involved.

Autophagy is a dynamic process; expression levels of LC3II/LC3I and SQSTM1/p62 indicate the magnitude of autophagic activity and flux (24). Increased expression of LC3II/LC3I and decreased expression of SQSTM1/p62 indicated a smooth autophagic flux; alternatively, the autophagic flux is inhibited (25). The LC3II/LC3I ratio had an upregulated trend during the first 6 hpi but was subsequently downregulated. Therefore, we inferred that autophagy was initially activated and the autophagic flux was smooth during the first 6 hpi, whereas the autophagic flux was subsequently inhibited at 8 to 12 hpi. During autophagosome formation, SQSTM1/p62 acts as a bridge linking LC3 and polyubiquitinated proteins, and expression levels of SQSTM1/p62 protein are negatively correlated with autophagy activity (26, 27). In this study, protein expression of SQSTM1/p62 was downregulated at 6 hpi, indicating that autophagy activity was normal. However, upregulation of SQSTM1/p62 protein at 8 to 12 hpi implied that autophagy was subsequently inhibited. Furthermore, the autophagic flux can be monitored by expression of mCherry-GFP-LC3B fusion protein from adenovirus (28). Using this approach in the present study, the number of green spots and red spots increased gradually, especially at the first 6 hpi, consistent with the autophagic flux being unobstructed. However, at 8 to 12 hpi, the autophagic flux was inhibited. Autophagosome formation is closely related to clearance of intracellular aggregated proteins, damaged organelles and invading pathogens. In cells infected with *P. bovis* and treated with rapamycin, autophagosomes were confirmed by transmission electron microscopy. However, we did not observe *P. bovis* within the autophagosome, perhaps due to the invading *P. bovis* having been cleared. Therefore, *P. bovis* infection induced autophagy in bMECs through upregulation of the LC3II/LC3I ratio and downregulation of SQSTM1/p62 protein expression.

Initiation of autophagy is regulated by a variety of intracellular signaling pathways. The AMPKα/mTOR signal pathway is the critical factor regulating autophagosome formation (29, 30). Furthermore, mTOR is a serine/threonine protein kinase in the mTORC1 complex and a major regulator of autophagy (31). Activation of AMPK may cause autophagy by negatively modulating mTOR (32). *Prototheca bovis* infection upregulated protein expression of p-mTOR, whereas rapamycin treatment decreased p-mTOR protein expression in bMECs. Furthermore, protein expression of autophagy-related genes, including Atg5, Beclin1 and LC3II/LC3I (but not SQSTM1/p62), were also upregulated in bMECs, indicating the occurrence of autophagy. High expression of p-mTOR protein may be related to AMPKα, as protein expression of AMPKα and p-AMPKα were upregulated in bMECs. Although activation of AMPKα did not negatively modulate mTOR, autophagy in bMECs may be linked to the ability of AMPK to directly phosphorylate ULK1 ser 317 and induce autophagy. *Prototheca bovis* infection upregulated protein expression of ULK1 and p-ULK1 (ser 317) in bMECs. Expression of ULK1 is also regulated by mTOR-dependent inhibitory phosphorylation under nutrient-rich conditions (33). Under nutrient sufficiency, high mTOR activity prevents ULK1 activation by phosphorylating ULK1 ser 757 and disrupting the interaction between ULK1 and AMPK. In contrast, under pathological conditions (infection or drug treatment), elevated phosphorylated ULK1 (ser 757) protein initiates autophagy, and high mTOR activity does not completely prevent ULK1 (ser 757) phosphorylation to inhibit autophagy in cells (33, 34). *Prototheca bovis* infection increased protein expression of ULK1 and p-ULK1 (ser 757) in bMECs, whereas mTOR did not completely inhibit autophagy by phosphorylating ULK1 ser 757. We inferred that activation and phosphorylation of ULK1 were not only related to activation of AMPKα, but also to elevation of mTOR, as there was no negative regulation between AMPKα and mTOR in bMECs. Therefore, activation of protein in the AMPKα/ULK1 pathway was involved in *P. bovis* infection-induced autophagy in bMECs.

**Figure 8:**
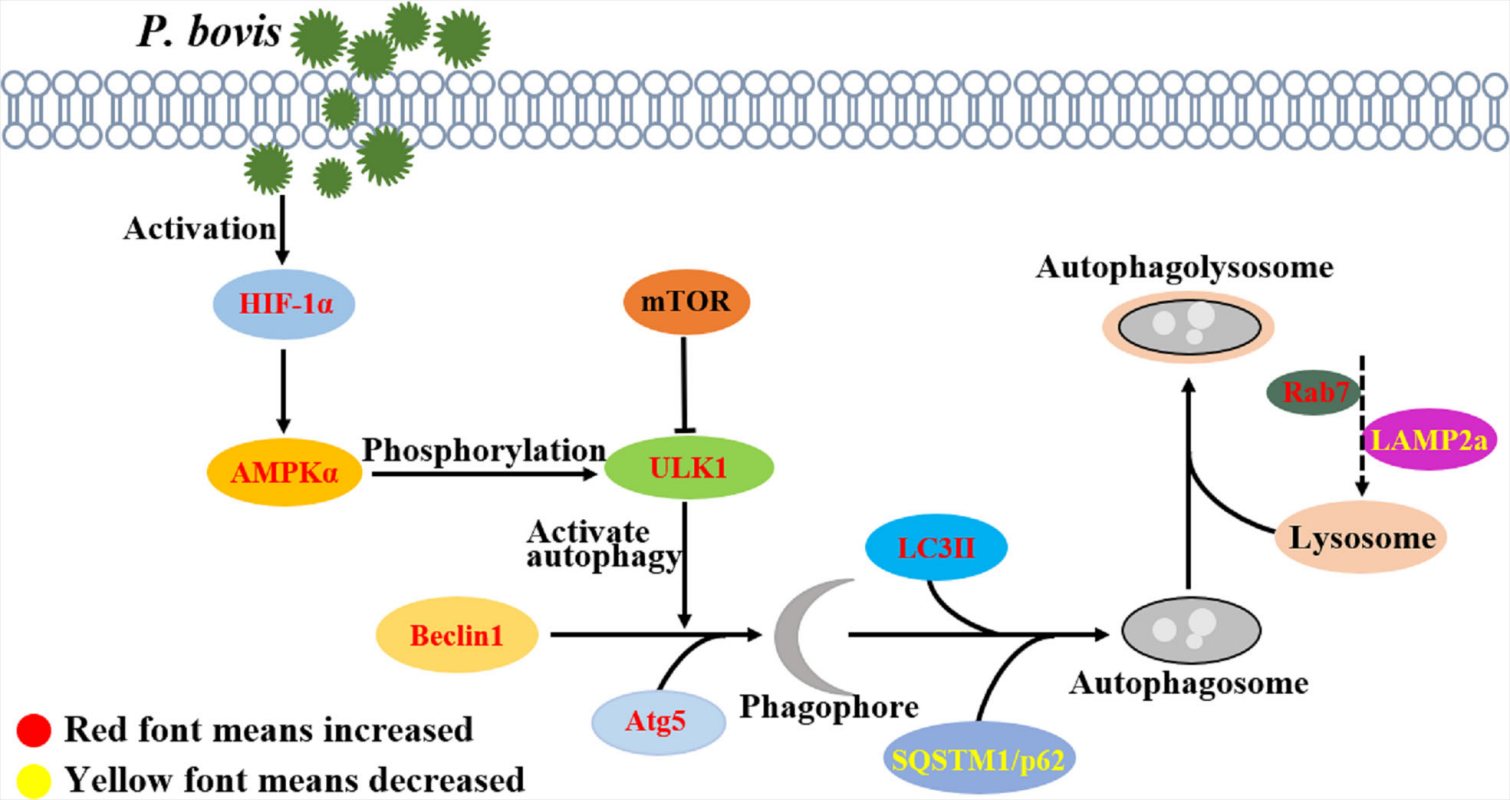
P*. bovis* infection induced autophagy in bMECs. *P. bovis* infection activates HIF-1α, and activation of HIF-1α has an important role in regulating AMPKα and ULK1, whereas activation of AMPKα promotes ULK1 phosphorylation. During *P. bovis* infection, ULK1 activates autophagy, and Beclin1 and Atg5 are involved in phagophore formation. Then, LC3II and SQSTM1/p62, as an indicator of autophagic activity, are involved in autophagosome formation. *P. bovis* infection induces autophagy *via* the HIF-1α and AMPKα/ULK1 pathway, and Rab7 and LAMP2a are involved in autophagolysosome formation induced by *P. bovis* in bMECs.

Activation of ULK1 is considered a key regulator of autophagy, with AMPK and mTOR catalyzing activation of ULK1 (35, 36). In the current study, *P. bovis* infection contributed to AMPKα, mTOR, and ULK1 phosphorylation and induced autophagy in bMECs. However, deletion of AMPKα decreased protein expression of ULK1 and p-ULK1 in *P. bovis* infected bMECs, implicating AMPKα as having an important role in activation of ULK1 and promoting ULK1 phosphorylation. Meanwhile, protein expression of SQSTM1/p62 was downregulated in bMECs, indicating that AMPKα deletion did not affect SQSTM1/p62 expression. In addition, protein expression of LC3II/LC3I was downregulated and the number of LC3 puncta reduced in bMECs, indicating that *P. bovis* infection induced autophagy was inhibited in bMECs. AMPKα deletion reduced ULK1 phosphorylation, but protein expression of HIF-1α was not decreased compared to the *P. bovis* infection group, suggesting that AMPKα may be a downstream factor of HIF-1α. Silencing AMPKα reduced autophagy induced by *P. bovis* infection in bMECs. Therefore, AMPKα has an important role in autophagy activation by promoting ULK1 phosphorylation in bMECs infected with *P. bovis*. ULK1 acts as a bridge between upstream nutrient or energy sensors mTOR and AMPK and downstream autophagosome formation, and phosphorylated ULK1 is considered a key regulator of autophagy (37). In the current study, deletion of ULK1 upregulated SQSTM1/p62 protein expression and downregulated LC3II/LC3I protein expression in bMECs infected with *P. bovis*, which indicated that autophagy induced by *P. bovis* infection was inhibited. Furthermore, the reduced number of LC3 puncta also indicated that autophagy was inhibited due to ULK1 deletion in bMECs. Additionally, deletion of ULK1 did not decrease protein expression of AMPKα and HIF-1α compared to the *P. bovis* infection group, suggesting that ULK1 may be a downstream factor of AMPKα. Therefore, we concluded that *P. bovis* infection induced autophagy by modulating the AMPKα/ULK1 pathway in bMECs (**Figure 8**). As the regulatory subunit of hypoxia-inducible factor 1 (HIF-1), HIF-1α is the master regulator of oxygen homeostasis (38, 39). Furthermore, it is also involved in many cellular processes, including inflammatory response, apoptosis, and autophagy (40–42). Deficiency of HIF-1α enhances influenza A virus replication by promoting autophagy in alveolar type II epithelial cells (22). In the present study, *P. bovis* infection upregulated HIF-1α protein expression in bMECs. In addition, silencing HIF-1α decreased protein expression of AMPKα and ULK1 in bMECs during *P. bovis* infection, whereas SQSTM1/p62 protein expression was upregulated, suggesting that AMPKα and ULK1 may be a downstream factor of HIF-1α and HIF-1α may indirectly affect expression of ULK1 by regulating AMPKα in bMECs. Therefore, we inferred that HIF-1α deletion inhibited autophagy in bMECs induced by *P. bovis*. Furthermore, we concluded that HIF-1α has an important role in autophagy induced by *P. bovis* through regulated AMPKα and ULK1 protein expression in bMECs (**Figure 8**).

Lysosomes degrade cytoplasmic proteins and damaged organelles, as well as autophagolysosomes formed during autophagy (43). Furthermore, Rab7 is essential for autophagosome-lysosome fusion during in the last phases of autophagy, with downregulation of Rab7 causing an autophagic block (13, 44). Infection with *P. bovis* upregulated protein expression of Rab7 in bMECs at 6 hpi, but downregulated it at 12 hpi. We concluded that autophagy was normal at 6 hpi but blocked at 12 hpi, and furthermore, that changes in Rab7 causing autophagy were consistent with previous studies. Additionally, lysosome-associated membrane protein 2a (LAMP2a) is a component of the lysosomal membrane, with an important role in maintaining structural integrity of the lysosome (13, 45). *Prototheca bovis* infection downregulated protein expression of LAMP2a in bMECs at 6 hpi, but upregulated it at 12 hpi, albeit neither change was significant, suggesting that lysosomes were normal at 6 hpi but damaged at 12 hpi, and that this lysosomal damage prevented autophagy. Abnormal fusion of mature lysosomes and autophagosomes will severely affect the clearance of damaged organelles and pathogens, and autophagy (confirmed by lysosomal staining). Therefore, we concluded that lysosome-associated protein Rab7 and LAMP2a had important roles in *P. bovis*-induced autophagy in bMECs.

In conclusion, *P. bovis* infection is one of the main causes of mastitis in dairy cows, and autophagy is an effective way to defense against pathogenic infection. *P. bovis* infection activated HIF-1α and promoted expression of AMPKα and ULK1; knocking down either of them inhibited autophagy in bMECs. This was interpreted as evidence that *P. bovis* infection induces autophagy in bMECs *via* the HIF-1α and AMPKα/ULK1 pathway.

## Data availability statement

The original contributions presented in the study are included in the article/**Supplementary Material**. Further inquiries can be directed to the corresponding authors.

## Author contributions

BH and WZ conceived and designed the experiment. WZ, MX, XX and ZD performed the research and wrote the manuscript. HB, DW, YL, and SK assisted in the analyses and re-edited the manuscript. HB, JK and BH revised the manuscript. All authors read and approved the final manuscript.

## Funding

Financial support for this study was received from the following: Beijing Municipal Natural Science Foundation (no. 6222031), Ningxia Key R&D Project (No. 2019BBF02027), the National Natural Science Foundation of China (No. 31572587, 31760751), the High-End Foreign Experts Recruitment Program (No. GDT20171100013) and the Natural Science Foundation of Ningxia (No. 2022AAC02022).

## Conflict of interest

The authors declare that the research was conducted in the absence of any commercial or financial relationships that could be construed as a potential conflict of interest.

## Publisher’s note

All claims expressed in this article are solely those of the authors and do not necessarily represent those of their affiliated organizations, or those of the publisher, the editors and the reviewers. Any product that may be evaluated in this article, or claim that may be made by its manufacturer, is not guaranteed or endorsed by the publisher.
