# Supplementary material for: Prototheca bovis induces autophagy in bovine mammary epithelial cells via the HIF-1α and AMPKα/ULK1 pathway
Source: Front Immunol. 2022 Sep 2;13:934819. doi: 10.3389/fimmu.2022.934819 (PMC9486811; doi:10.3389/fimmu.2022.934819)
Supplement: Supplementary file 1 [file DataSheet_1.docx]

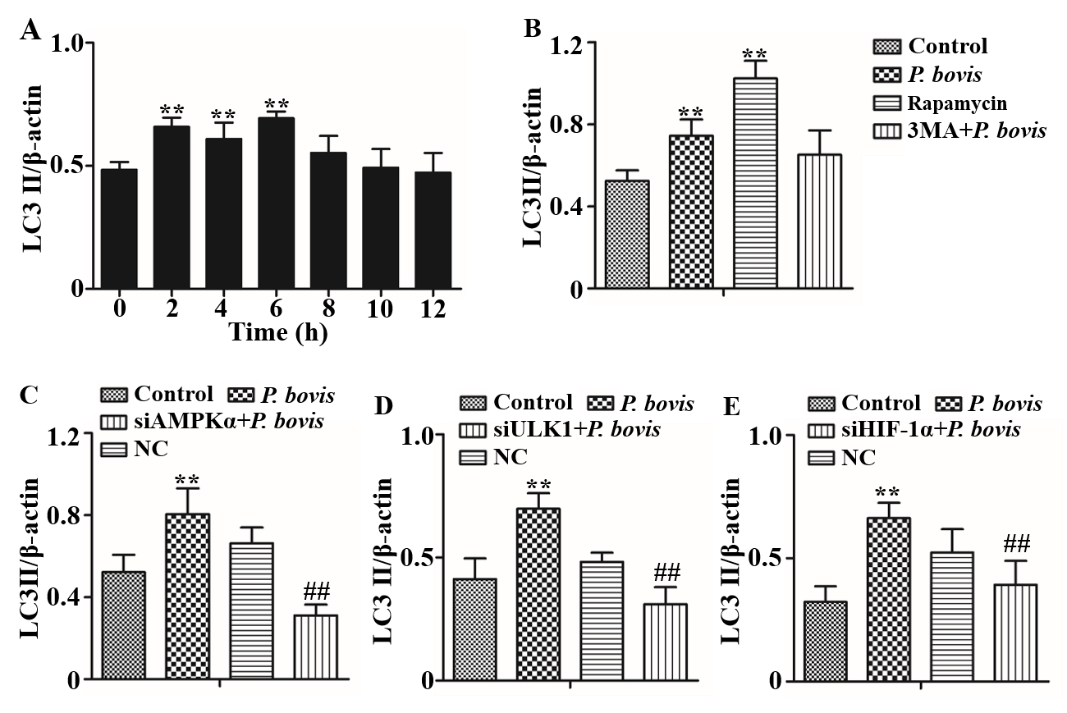


**Figure 1S Quantification of the LC3II band relative to β-actin in bMECs.** (A) Protein expression of LC3II in bMECs continuously infected for 12 h. (B) Western blot analyses protein expression of LC3II in bMECs. (C) Effects of silencing AMPKα on protein expression of LC3II in bMECs. (D) Effects of ULK1 deletion on protein expression of LC3II in bMECs. (E) Effects of silencing HIF-1α on protein expression of LC3II in bMECs. The right panel indicates protein quantification by ImageJ software. Data represent means ± SD of 3 independent experiments, compared to the control group (*P* < 0.05).


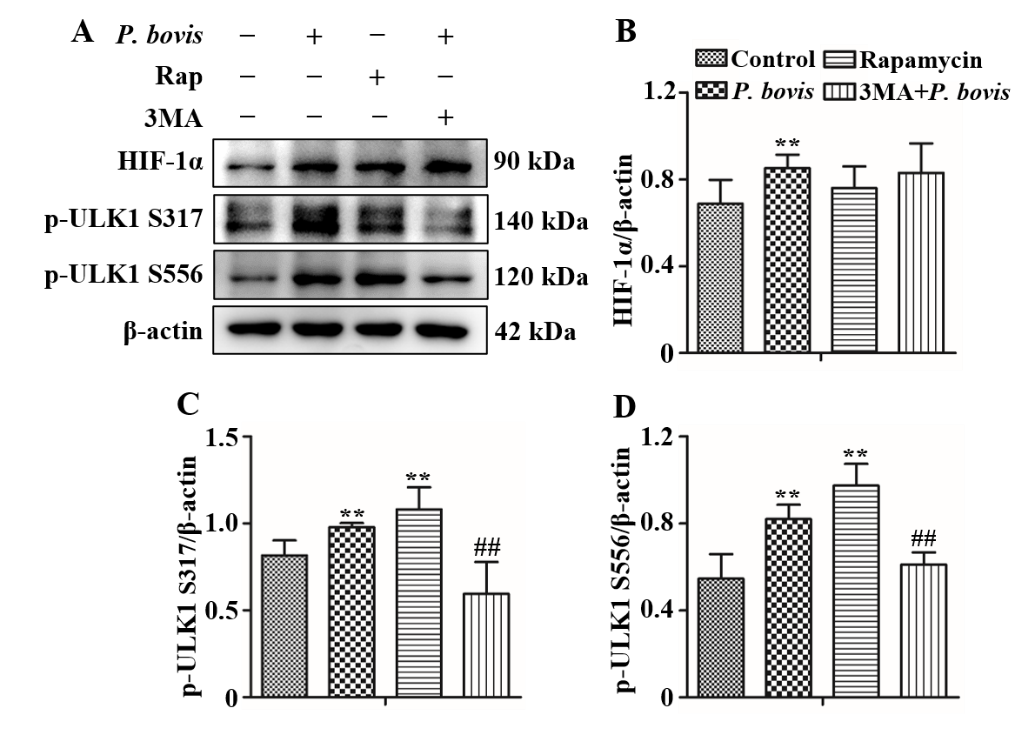


**Figure 2S The change in expression of HIF-1α protein in bMECs.** (A), (B), (C) and (D) Western blot analyses of HIF-1α, p-ULK1 S317 and p-ULK1 S556 in bMECs. The right panel is protein quantification with ImageJ software. The bMECs were infected with *P. bovis* and treated with rapamycin (20 µM) for 6 h. Additionally, bMECs were pretreated with 3MA (5 mM) for 2 h, and then infected with *P. bovis* for 6 h. Data represent means ± SD of 3 independent experiments, compared to the control group (*P* < 0.05).


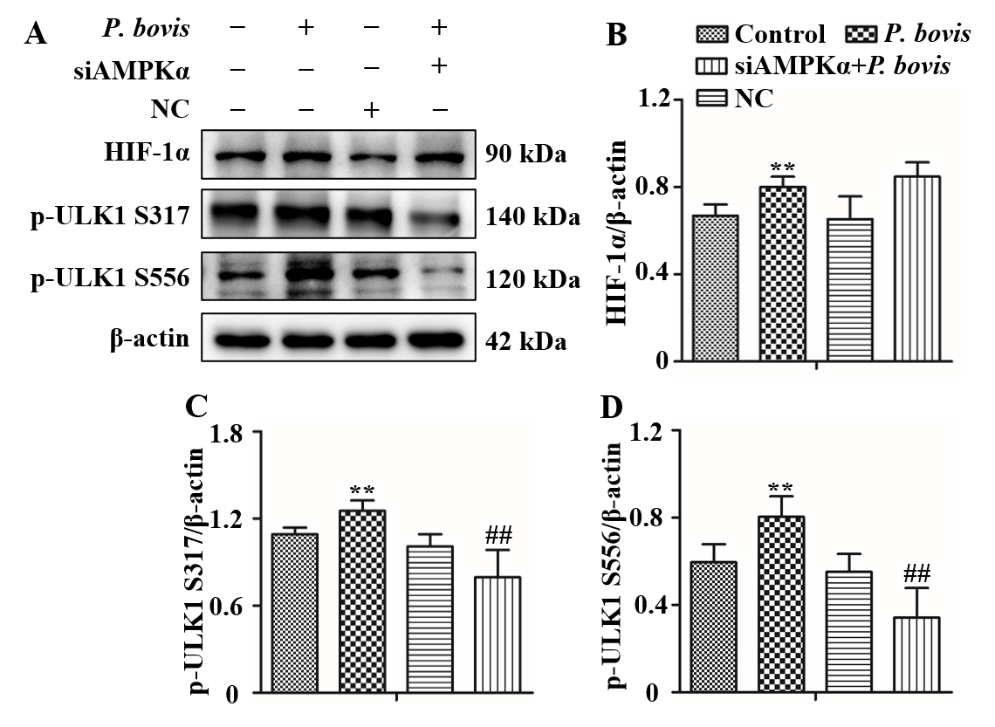


**Figure 3S Silencing AMPKα reduced *P. bovis*-induced autophagy in bMECs**. (A), (B), (C) and (D) Western blot analysis of HIF-1α, p-ULK1 S317 and p-ULK1 S556 in bMECs. The right panel indicates protein quantification (ImageJ software). Data represent means ± SD of 3 independent experiments, compared to the control group (*P* < 0.05).


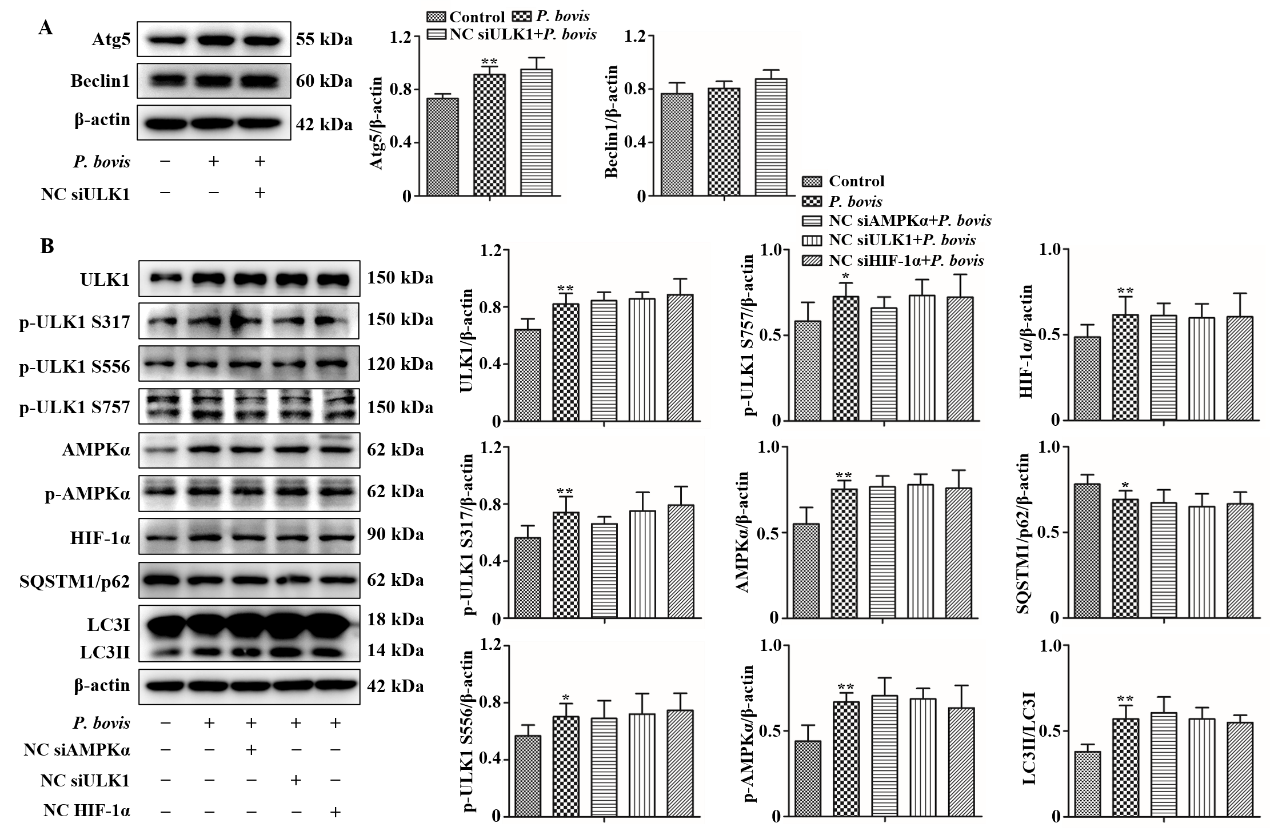


**Figure 4S Effects of non-target siRNA on protein expression of autophagy-related genes in *P. bovis*-infected bMECs.** (A) Western blot analyses of Atg5 and Beclin1 after treatment non-target siRNA (NC siULK1) in *P. bovis*-infected bMECs. (B) Western blot analyses of ULK1, p-ULK1, AMPKα, p-AMPKα, HIF-1α, SQSTM1/p62 and LC3II/LC3I after treatment non-target siRNA (NC siULK1, NC siAMPKα and HIF-1α) in *P. bovis*-infected bMECs. The right panel indicates protein quantification by ImageJ software. Data represent means ± SD of 3 independent experiments, compared to the control group (*P* < 0.05).


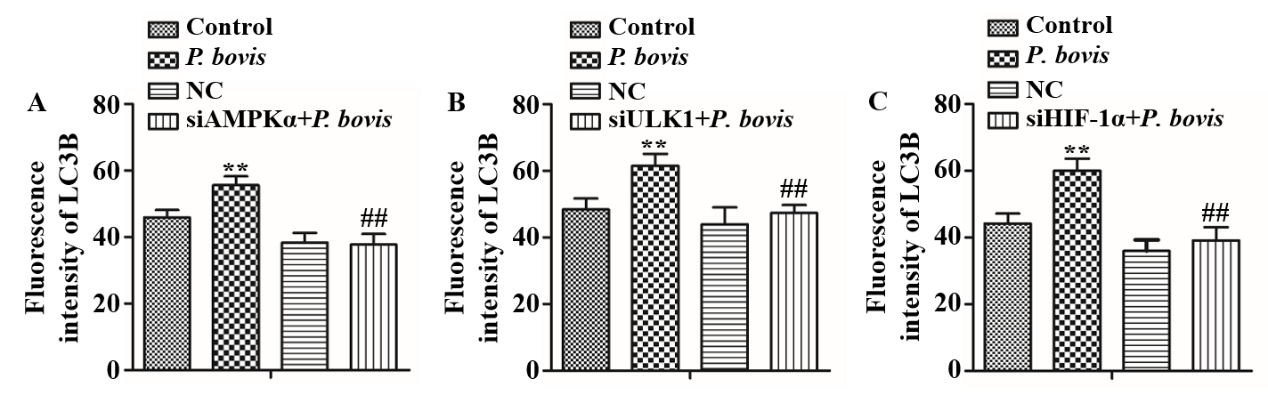


**Figure 5S The fluorescence intensity of total GFP-LC3B in bMECs.** (A), (B) and (C) The changes of total GFP-LC3B fluorescence intensity during *P. bovis* infection or deletion of AMPKα, ULK1 and HIF-1α in bMECs. Fluorescence intensity was quantified by ImageJ software. Data represent means ± SD of 3 independent experiments, compared to the control group (*P* < 0.05).


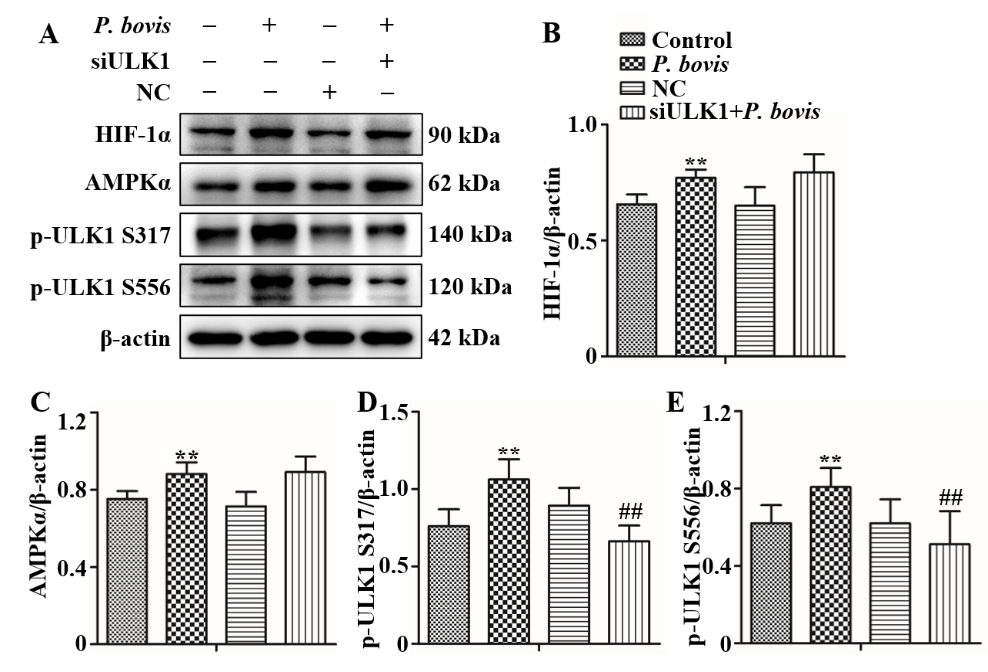


**Figure 6S Silencing ULK1 reduced *P. bovis* induced autophagy in bMECs.** (A), (B) and (C) Western blot analyses of HIF-1α, AMPKα, p-ULK1 S317 and p-ULK1 S556 after *P. bovis* infection for 6 h in bMECs. The right panel is protein quantification by ImageJ software. Data represent means ± SD of 3 independent experiments, compared to the control group (*P* < 0.05).


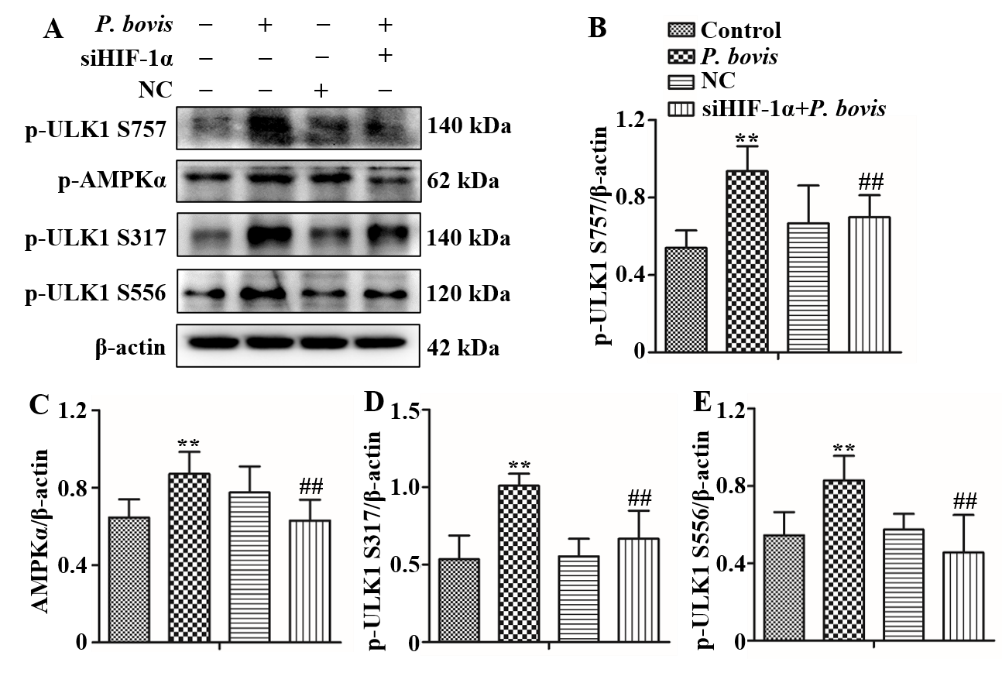


**Figure 7S HIF-1α deletion reduced *P. bovis* induced autophagy in bMECs.** (A), (B) (C), (D) and (E) Western blot analyses of p-AMPKα, p-ULK1 S757, p-ULK1 S317 and p-ULK1 S556) in bMECs after *P. bovis* infection for 6 h. The right panel indicates protein quantification by ImageJ software. Data represent means ± SD of 3 independent experiments, compared to the control group (*P* < 0.05).
